# Adhesion Properties of Lactic Acid Bacteria on Intestinal Mucin

**DOI:** 10.3390/microorganisms4030034

**Published:** 2016-09-20

**Authors:** Keita Nishiyama, Makoto Sugiyama, Takao Mukai

**Affiliations:** 1 Department of Microbiology, School of Pharmacy, Kitasato University, Tokyo 108-8641, Japan; nishiyamak@pharm.kitasato-u.ac.jp; 2 Faculty of Veterinary Medicine, School of Veterinary Medicine, Kitasato University, Aomori 034-8628, Japan; masugi@vmas.kitasato-u.ac.jp; 3 Department of Animal Science, School of Veterinary Medicine, Kitasato University, Aomori 034-8628, Japan

**Keywords:** adhesion, carbohydrate, colonization, gastrointestinal tract, histochemistry, lactic acid bacteria, mucin

## Abstract

Lactic acid bacteria (LAB) are Gram-positive bacteria that are natural inhabitants of the gastrointestinal (GI) tracts of mammals, including humans. Since Mechnikov first proposed that yogurt could prevent intestinal putrefaction and aging, the beneficial effects of LAB have been widely demonstrated. The region between the duodenum and the terminal of the ileum is the primary region colonized by LAB, particularly the *Lactobacillus* species, and this region is covered by a mucus layer composed mainly of mucin-type glycoproteins. The mucus layer plays a role in protecting the intestinal epithelial cells against damage, but is also considered to be critical for the adhesion of *Lactobacillus* in the GI tract. Consequently, the adhesion exhibited by lactobacilli on mucin has attracted attention as one of the critical factors contributing to the persistent beneficial effects of *Lactobacillus* in a constantly changing intestinal environment. Thus, understanding the interactions between *Lactobacillus* and mucin is crucial for elucidating the survival strategies of LAB in the GI tract. This review highlights the properties of the interactions between *Lactobacillus* and mucin, while concomitantly considering the structure of the GI tract from a histochemical perspective.

## 1. Introduction

The gastrointestinal (GI) tract of humans and other mammals is inhabited by ~10^14^ bacteria from approximately 1000 or more different species, which continuously interact with their host as they grow and form a diverse microbiota [1,2,3]. Of the human GI microbiota, relatively few bacteria inhabit the stomach, with <10^4^ bacteria present per g of stomach content, whereas this number increases further down the GI tract, with 10^5^–10^8^ and 10^11^ bacteria inhabiting the small and large intestines, respectively [4,5]. These bacteria produce myriad metabolites, which are frequently recognized as stimulating factors by the host. Accordingly, the behavior of the microbiota markedly influences the overall health of the host [6,7]. In recent years, whole-genome sequencing of bacteria and metagenomic analyses of microbiota performed using comprehensive genomic analytical techniques have revealed the structure of the symbiotic relationship between the host and the bacteria within the complex ecosystem that is the human body [8,9].

As a form of deliberate administration, certain bacteria can be proactively consumed to directly or indirectly improve the microbiota, and this shows considerable potential for producing health-maintaining effects in humans and animals. These bacteria are frequently referred to as probiotics, and these organisms have been defined by the International Scientific Association for Probiotics and Prebiotics consensus statement based on the FAO/WHO Guidelines as “a live microorganism that, when administered in adequate amounts, confers a health benefit on the host” [10].

Lactic acid bacteria (LAB), which include *Lactobacillus* species, produce lactic acid (>50% of sugar carbon) as the main end product of carbohydrate metabolism. Taxonomically, the genus *Lactobacillus* belongs to the phylum Firmicutes, class Bacilli, order *Lactobacillales*, and family *Lactobacillaceae* *Lactobacillus* comprises a large heterogeneous group of low-G + C Gram-positive, non-sporulating, anaerobic bacteria [11] that includes over 145 recognized species [12]. *Lactobacillus* species are present throughout the GI tract of mammals, such as humans, pigs, hamsters, mice, rats, dogs, sheep, and cattle, and also several birds [13]. Moreover, *Lactobacillus* species are frequently detected as dominant bacteria in the region from the duodenum to the terminal of the ileum in the human gut [14] and in the female urogenital tract [15,16], but are present in a very low proportion (<10^4^ bacterial counts) in the colon [15]. By contrast, rodents and chickens harbor comparatively large numbers of lactobacilli in the upper gut and the forestomach and crop, respectively [17].

The mucus layer covering the GI tract is the first point of contact between the intestinal microbiota and the host, and it provides a habitat for the microbiota [18,19]. Moreover, the secretion and turnover of mucin and the movement of gut contents create a fluid environment in the GI tract. Therefore, adhesion to the mucosal surface is one of the critical prerequisites for the colonization of non-motile organisms in the GI tract, which also provides the organisms a competitive advantage in this ecosystem [20,21,22]. In the last 10 years, the availability of a complete repertoire of tools for *Lactobacillus* genetics and the advancement of analytical techniques have markedly accelerated research on the molecular mechanisms used by *Lactobacillus* strains to recognize and adhere to mucin. Such mechanisms involve carbohydrate-protein interactions with the mucin chain through diverse adhesins associated with the bacterial cell surface, as previously described in several reviews [23,24,25]. Here we provide an updated review of the field while concurrently considering the structure of the GI tract mucosa from a histochemical perspective.

## 2. Anatomy and Histology of the Mouse and Human GI Tracts

**Figure 1:**
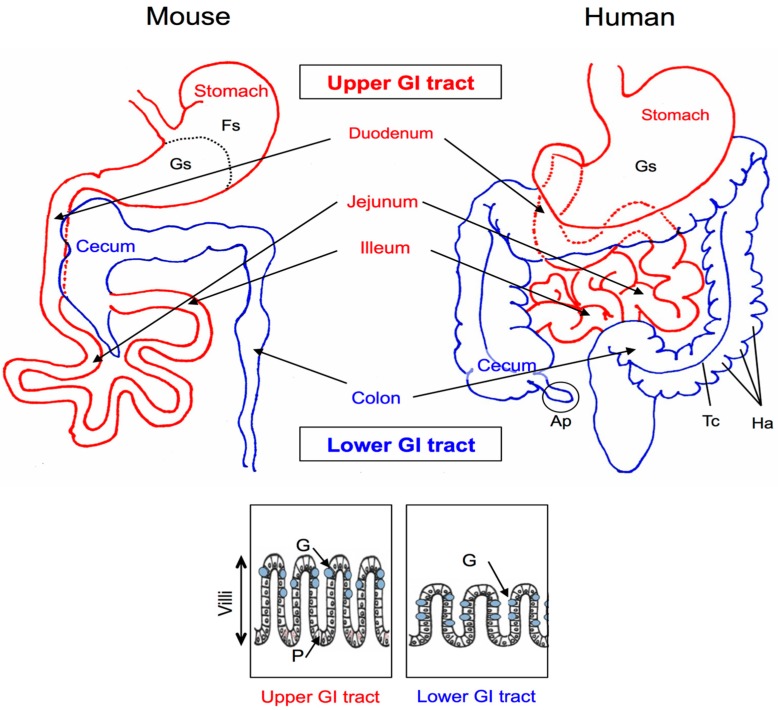
Mouse and human abdominal anatomy. The gastrointestinal (GI) tract is divided into two regions: the upper (red) and the lower (blue) GI tract. The rectangles show schematic models of the villi of the upper and lower GI tracts. G, goblet cells; P, Paneth cells; Fs, forestomach; Gs, glandular stomach; Ap, appendix; Tc, taenia coli; Ha, haustra.

The mammalian GI tract can be divided into two main parts: the upper and the lower GI tract (Figure 1). The primary functions of the upper GI tract are enzymatic digestion, absorption of nutrients, and protection against the external environment. The primary function of the lower GI tract is to dehydrate and store fecal material. The GI tracts of monogastric animals are similar with respect to their general structures and functions. However, various anatomical differences in the gut structures exist between animal species, and these differences create a host-specific GI environment [1]. Because murine species (particularly mice) are commonly used as an in vivo experimental colonization model [22,26,27,28], in this section, we describe the structure of the GI tract mucosa from a histochemical perspective with comparison of the human and murine intestinal systems.

Among the structures of the upper GI tract, the stomach shows the greatest gross anatomical difference between mice and humans (Figure 1); whereas the murine stomach is grossly divided into two distinct regions, the forestomach and the glandular stomach, humans lack the forestomach [29,30]. In the small intestine of mice, the villi are taller than those in humans, and this provides an increased surface area to compensate for the lack of plicae, which are found in the human small intestinal structure [30]. In the lower GI tract of mice, a large cecum is present and an appendix is absent (Figure 1, left), and whereas the human colon contains the taenia coli and haustra (Figure 1, right), the mouse colon features a smooth serosal line [31]. The mouse cecum is a functional fermentation organ that contains a high population of bacteria, and mice can readily capture the free fatty acids and vitamins produced by these bacteria. These anatomical differences between the human and mouse GI tracts exert a mechanical and spatial influence on the colonization conditions, which results in the aforementioned development of host-specific gut microbiota [1,17].

Paneth cells and goblet cells are the principal secretory cells of the intestinal epithelium. Paneth cells of the small intestinal crypts are the main source of antibacterial peptides [32,33]. Goblet cells are present in the GI epithelia of mammals, and these cells produce a large amount of the mucus (mucin) that coats the intestinal epithelium. This mucus layer and the gastric mucosa lining the gut epithelia contain antimicrobial peptides, cytokines, and immunoglobulins [34]. Therefore, both cell types secrete products that regulate the innate and adaptive immune systems in the GI tract. Goblet and Paneth cells are conserved between mice and humans, although their distribution shows differences: in mice, goblet cells are prominent along the surface of the intestinal crypts in the proximal colon, but their numbers are decreased in the distal colon and rectum at the base of the crypt. Conversely, in humans, goblet cells are consistently abundant from the cecum to the rectum [35]. Paneth cells in mice are most abundant in the jejunum and are not detected in the large intestine (see schematic models in Figure 1, lower) [29], whereas Paneth cells in humans are normal constituents of the small intestine, appendix, and cecum [36]. In mice, goblet cells are the main type of mucus-producing cells and are most prevalent in the ileum. In addition to these local differences, the amounts of peptides or mucus secreted from and stored in Paneth cells differ between mice and humans [29].

## 3. Histochemical Studies of the GI Mucus

Mucin glycoproteins are the most prevalent structural components of the GI mucus, followed by a complex mixture of lipids, enzymes, nucleic acids, and secretory immunoglobulin A. Furthermore, because water weight accounts for at least 95% of the GI mucus [37], observing the detailed morphology of the mucus layer is challenging. Therefore, optimal preservation, embedding, and proper selection of the fixative used are the first essential steps for successful histochemistry.

Two main fixatives are commonly used for histochemical purposes: (i) crosslinking fixatives (e.g., formaldehydes such as neutral-buffered formalin or paraformaldehyde) and (ii) denaturing fixatives (e.g., alcohol-based fixatives such as Carnoy’s fixative) [38]. Formaldehyde acts by forming intramolecular and intermolecular crosslinks, including ethoxylated adducts, crosslinked molecules with amines, and depurination fragments from nucleic acids [39]. Conversely, alcohol solutions cause protein dehydration, which results in protein coagulation and tissue shrinkage. In the case of Carnoy’s fixative, chloroform and acetic acid are added to the mixture, which prevents the shrinkage effects of ethanol, and tissues are fixed through hydrogen bonding of the constituents to the tissue [40]. Methacarn is a fixative in which methanol is used instead of ethanol in Carnoy’s solution to counteract the shrinkage effect [41]. To optimize visualization of the natural mucin distribution of the human colon, Ota and Katsuyama [42] evaluated paraffin sections embedded with 5 distinct fixatives (3 crosslinking fixatives: phosphate-buffered 10% formalin, Bouin’s solution, and HgCl_2_-glutaraldehyde solution; and 2 denaturing fixative: Carnoy’s solution and 100% ethyl alcohol); only the Carnoy fixative yielded satisfactory results.

**Figure 2:**
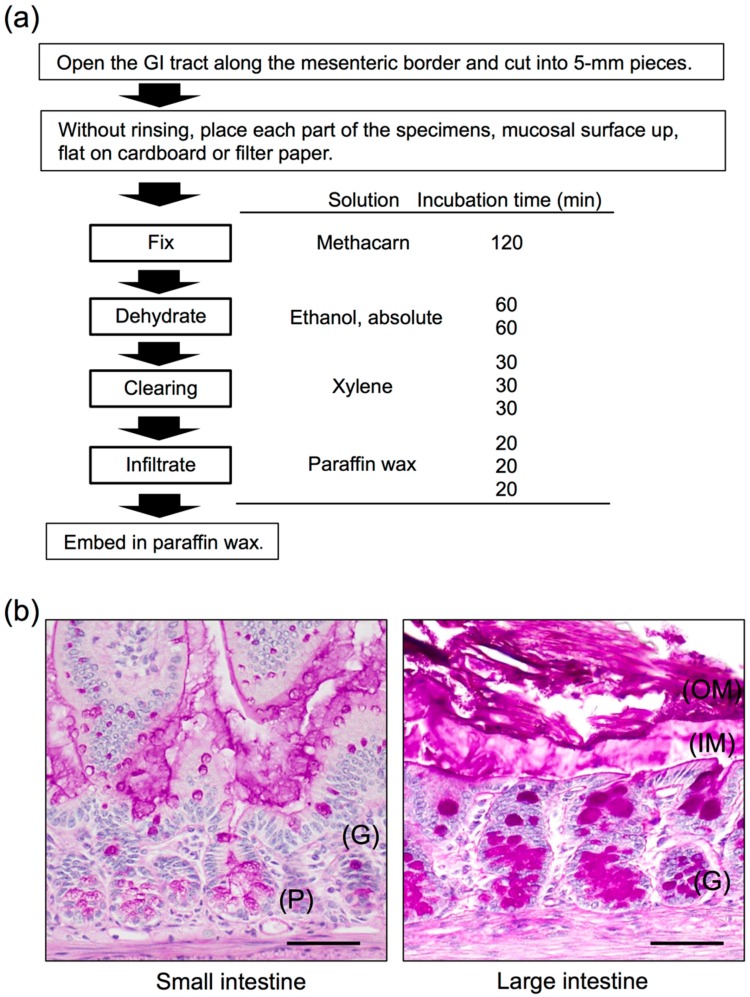
Histology of the mouse small and large intestines. **(a)** Methacarn fixative and paraffin embedding procedure and methods, based on Puchtler’s protocol [41]. **(b)** Transverse sections of a 12-week-old male mouse small intestine (jejunum) and large intestine (colon). Methacarn fixative, periodic acid-Schiff stain. OM, outer mucus; IM, inner mucus; G, goblet cells; P, Paneth cells. Scale bars: 100 µm. Based on the results of Nishiyama et al. [47].

Figure 2a shows our suggested protocol for histochemical studies of the mucus of the GI tract, which is based on the method of Puchtler et al. [41] and includes several modifications of the solutions and processing times. The key procedural steps are to immerse the specimen in the fixative solution immediately before autolysis and degradation, and to prevent tissue shrinkage. As shown by the examples in Figure 2b, by using this method, we successfully observed highly conserved mucus layers in murine jejunum and colon sections. Notably, the mucus layer showed weakly positive periodic acid-Schiff (PAS) staining (outer mucus layer), whereas the high-density mucus layer that contacts epithelial cells showed strong PAS-positive staining (inner mucus layer). Such a double mucus layer structure is characteristic of the large intestine. By contrast, the small intestine formed a single layer of mucus attachment, which showed only weakly positive PAS staining (Figure 2b). In mice, the mucus gel layer in the small and large intestines is approximately 100–400-μm thick, with the thickest section detected in the duodenum [43,44]. Moreover, Johansson et al. [18] analyzed colonic tissue sections for bacterial presence through in situ hybridization performed using a general 16S rRNA probe. Microbiota colonized the outer mucus layer, but could not pass through the inner mucus layer bordering epithelial cells, which is packed with densely linked mucin. Therefore, whereas the inner mucus layer plays the key role of preventing bacterial contact with and invasion into epithelial cells, the expanded outer mucus layer is a major habitat for commensal bacteria [18].

## 4. Mucin Biosynthesis

**Figure 3:**
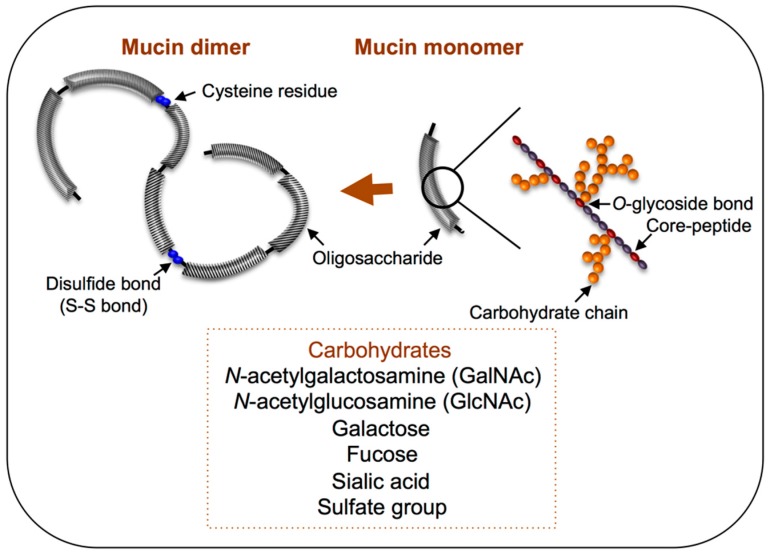
Polymeric structure of mucin molecules. A simplified scheme showing the composition of mucin glycoproteins, in monomer and dimer forms. Glycosylated regions are shown as black diagonal tubes, in which carbohydrate chains form a closely packed sheath around the central peptide core.

Mucin secretion is an extremely dynamic process, and new mucin is continuously produced in colonic goblet cells and features a turnover rate of <1 h [43]. The gene *muc* encodes the core protein of mucin, and approximately 20 different mucin-encoding genes have been discovered in humans to date, and a few homologous *muc* genes have been identified in other animal species [23,34]. Among the secreted mucins, the main gel-forming molecules are MUC2, MUC5AC, MUC5B, and MUC6 [45], and MUC2 is the predominant mucin in the small and large intestines [46]. As illustrated in Figure 3, the molecular structure of MUC2 mucin has been likened to a “bottle-brush”. Mucin-type *O*-glycans are covalently α-linked via an *N*-acetylgalactosamine (GalNAc) moiety to the hydroxyl group of serine or threonine residues within a peptide backbone (Figure 3). The transfer process of the glycosylation reaction occurs in the Golgi apparatus, starting with the addition of GalNAc, following which various carbohydrate chains are formed through the addition of galactose, *N*-acetylglucosamine (GlcNAc), fucose, or sialic acid, depending on the glycosyltransferase prevalence in the cell. In certain cases, sulfotransferases also add a sulfate group. After the carbohydrate chains have been added in the Golgi apparatus and the molecule is secreted, disulfide bonds form between all of the monomers at their N-terminal cysteine residues, which results in the assembly of a single giant polymer (Figure 3). Moreover, the human ABO blood-group substance is frequently expressed on the non-reducing terminal of the mucin glycan, which generates carbohydrate chains featuring diverse structures [46].

Histochemically, mucin can be classified as neutral or as acidic when its non-reducing terminal is modified by sialic acid or a sulfate group, which results in an overall negative charge [48]; therefore, these residues have been considered the target binding site for intestinal microbiota, as discussed in Section 5 and Section 6. Specifically, sialomucin and sulfomucin, which are modified by sialic residues and sulfate groups, respectively, show drastic changes according to host, age, and digestive organs and under certain conditions such as cancer and inflammatory bowel disease (IBD) [48,49]. Based on a histochemical study performed on human colonic biopsy samples, sialomucin and sulfomucin were both shown to increase gradually along the right colon to the rectum, whereas sulfomucin was predominant throughout the colon [50]. By contrast, in certain rodents (mice, rats, and guinea pigs), sialomucin was increased from the cecum to the distal colon, but sulfomucin appeared only in the distal colon [51]. In humans, the amount of acidic mucin has been found to be altered in IBD, where sulfomucin levels are decreased [52] and sialomucin is increased [53]. Interestingly, a previous study found increased proportions of *Lactobacillus* in patients with IBD [54]. Although the relationship with adhesion of *Lactobacillus* is not clear, modulation of mucin carbohydrates might affect host- or digestive-organ-specific *Lactobacillus* colonization.

Moreover, a recent report indicated that increased sulfomucin levels in human colonic tissues correlated with elevated representation of sulfate-reducing bacteria [50], which can obtain energy by oxidizing organic compounds or molecular hydrogen while concomitantly reducing sulfate to hydrogen sulfide. Therefore, these lines of evidence raise additional possibilities: the carbohydrates and terminal groups present on mucin oligosaccharide chains can serve both as binding sites and as a potential source of nutrients for GI microbes, and might contribute to the composition of host-specific microbial communities.

## 5. Interactions of *Lactobacillus* with Intestinal Glycoconjugates

A considerable amount of research has been conducted to deepen our understanding of the interaction between bacteria and intestinal glycoconjugates. In the case of pathogenic bacteria in particular, the step in which the bacterium adheres to the host is critical during the course of proliferation and infection inside the host. The concept of bacterial adherence to host carbohydrate chains was first proposed following observations of the interference of *Escherichia coli* adherence to cells upon the addition of mannose [55]. Moreover, the pili that cover *E. coli* were found to exhibit lectin-like properties, and the interactions between the bacterial adhesion factors and receptors through carbohydrate chains were revealed [56]. Subsequent research showed that *Helicobacter pylori* adhere using the heat-shock proteins Hsp70 and BabA, which are adhesion factors for the sulfated carbohydrate chains and blood-group antigens that are present in the gastric mucin of humans, and further that the interactions with carbohydrate chains are critical for *H. pylori* infection [57,58]. These findings then raised the question of what types of interactions might exist between *Lactobacillus* and intestinal glycoconjugates.

In the early 1990s, a *Lactobacillus* strain was identified in which adhesion to blood-cell coagulation and mucus-secreting HT29-MTX cells decreased after processing with a protease [59,60]. This was the first report to suggest the existence of an adhesion process in *Lactobacillus* that is similar to that used by pathogenic bacteria: an “extracellular bridging protein” interacts with a component of the bacterial cell and a receptor on the intestinal epithelium [60]. Following this discovery, the interaction between glycolipid carbohydrate chains and *Lactobacillus* was elucidated based on the results of thin-layer chromatography. *Lactobacillus casei* IFO3425 was shown to bind to carbohydrate chains that contained either a galactosyl or glucosyl residue on the non-reducing terminal [61]. Furthermore, by using asialo-GM1, *Lactobacillus reuteri* JCM1081 was shown to strongly bind to neutral carbohydrate chains harboring a galactosyl residue on the non-reducing terminal [62]. These *Lactobacillus* strains did not bind to acidic carbohydrate chains that contained sialic acid, which indicated the existence of specific interactions between *Lactobacillus* and carbohydrate chains rather than the involvement of electrostatic forces. An adhesion mechanism mediated by lectin-like proteins was proposed for *Lactobacillus* [63], which led to further research on their interaction with mucin through carbohydrate chains. Studies were also conducted using a carbohydrate probe from an ABO blood-group antigen that is present on the non-reducing terminal of mucin-type carbohydrate chains, and this resulted in the discovery of several *Lactobacillus* strains exhibiting adhesion that were capable of binding to human blood antigens; this revealed a mechanism for adhesion to mucin carbohydrate chains through blood-group antigens [64,65]. Moreover, certain *Lactobacillus* strains were discovered in which neuraminidase- or sulfatase-mediated enzymatic processing, as well as physical inhibition by barium chloride, reduced the adhesion toward human colonic mucin [66]. This suggests that acidic mucin carbohydrate chains that contain sialic acid residues and sulfate groups might contribute to the adhesion of *Lactobacillus* to mucin. The aforementioned studied have provided evidence for the specific interactions of *Lactobacillus* through carbohydrate chains, including the interactions of *Lactobacillus* with the complex web of carbohydrate chains in mucin.

## 6. Mucin Adhesion Factors and Adhesion Mechanisms in *Lactobacillus*

**Figure 4:**
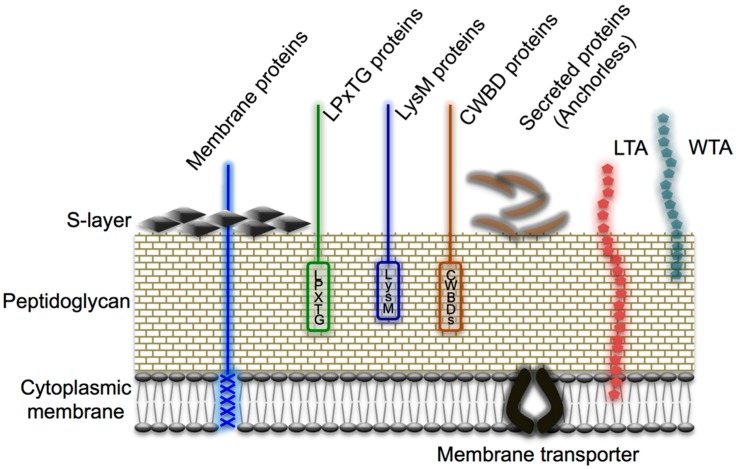
Cell-surface architecture of Gram-positive bacteria. A thick, multilayered peptidoglycan layer is decorated with lipoteichoic acid (LTA), wall teichoic acid (WTA), and various proteins, including S-layer proteins. Cell wall-anchored proteins are attached to the cell wall either (i) covalently by sortases (e.g., LPXTG proteins) or (ii) non-covalently (e.g., through a LysM motif or cell wall-binding domains [CWBDs]). Membrane proteins can covalently attach to the long-chain fatty acids of the cytoplasmic membrane, whereas S-layer proteins are attached to the cell wall through charged or uncharged secondary cell wall polymers. Reproduced from Desvaux et al. [67] with several modifications.

In this section, we discuss the molecular mechanisms involved in the adhesion exhibited by lactobacilli toward mucin. As shown in Figure 4, the surface of Gram-positive bacterial cells comprises a thick peptidoglycan layer, polysaccharides (e.g., teichoic acid and lipoteichoic acid), and various cell-surface proteins, including S-layer proteins. These are typical cell-surface structures of bacteria because the structures are in direct contact with the environment, and the cell-surface proteins in particular are involved in diverse physiological functions, and the proteins range from adhesion factors, antigens, and receptors to enzymes or transporters, as reviewed previously [21,67]. In Table 1, we list the major lactobacilli protein-like adhesion factors for mucin that have been reported to date. Adhesion factors possessing lectin-like properties have also been discovered in *Lactobacillus* strains that exhibit adhesion for blood-group antigens and acidic mucin carbohydrate chains, and this has facilitated the gradual elucidation of the adhesion mechanisms. Furthermore, the application of bioinformatics based on genomic data, the use of which has surged recently, and the development and availability of several new genetic tools have motivated further research directed toward uncovering the adhesion factors of *Lactobacillus* by using the sequence information stored in the surface proteins. Here, the adhesion factors for mucin and mucin carbohydrate chains are introduced. The cell-surface proteins that act as adhesion factors exhibit two main patterns of localization; these proteins include (i) the cell wall-anchored proteins that are covalently bonded to the cell wall by sortases through the anchor sequence (LPXTG) located at the C-terminus (Section 6.1), and (ii) multifunctional proteins that act as adhesion factors in addition to performing their primary intracellular functions (the so-called moonlighting proteins; Section 6.2).

### 6.1. Cell Wall-Anchored Proteins

**Figure 5:**
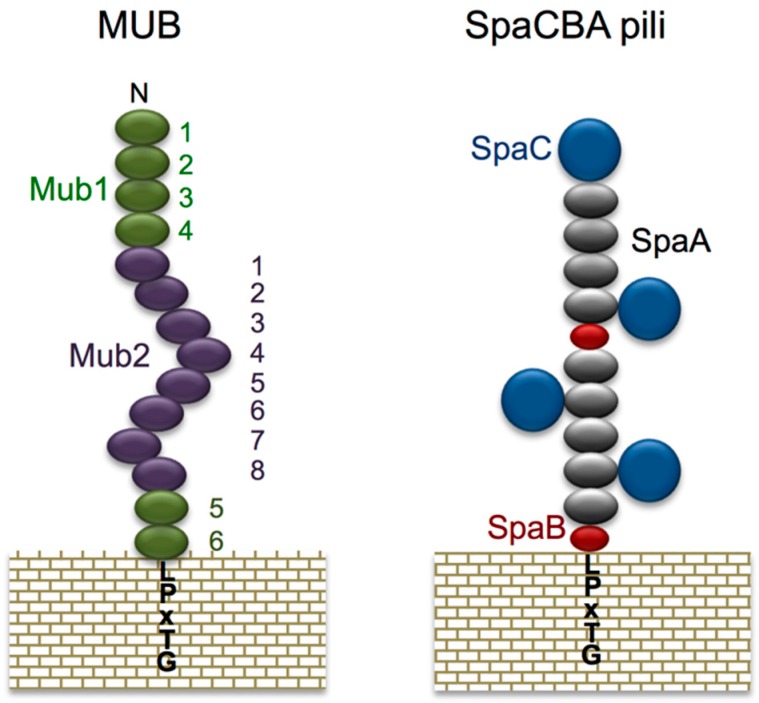
Schematic model of MUB and SpaCBA pili. MUB comprises 6 Mub1 repeats (green) and 8 Mub2 repeats (purple). The C-terminal LPXTG motif anchors MUB to the peptidoglycan of the bacterial cell wall. Reproduced from Roos and Jonsson [68] and Etzold et al. [70] with several modifications. The heterotrimeric SpaCBA pili are composed of shaft-forming SpaA (gray) major pilins together with SpaB (red) and SpaC (navy) minor pilins. Reproduced from Kankainen et al. [76] and Reunanen et al. [77] with several modifications.

Among the mucin adhesion factors of *Lactobacillus* identified thus far, the mucus-binding (MUB) protein family is probably the most extensively studied. First discovered in *L. reuteri* 1063 and *Lactobacillus acidophilus* NCFM, MUB is a large protein (molecular weight ≥ 300,000 Da) that features a characteristic YSIRK secretion signal sequence and a sortase-dependent LPXTG anchor motif conserved at its N- and C-termini, respectively [68,69]. MUB also contains two signature conserved regions of repeats named Mub1 and Mub2, with each repeat composed of 183–206 amino acid residues (Figure 5, left) [68,70]. In subsequent research, MUB’S role as a mucin adhesion factor in *L. reuteri* ATCC 53068 (same strain as *L. reuteri* 1063) was demonstrated through inhibition tests performed using anti-MUB antibodies [71]. The MUB repeat sequence was found to be highly similar to the mucin-binding protein (MucBP, PF06458) domain and an amino acid sequence reported in *Listeria monocytogenes* [72], which led to further investigation on MucBP homologous proteins in other bacterial species. To date, >10 bacterial species have been found to contain cell-surface proteins featuring amino acid sequences similar to those of the MucBP domain [70,71,73,74]. All of these proteins have been assumed to function as adhesins, but few investigations into their genuine roles have been conducted. However, recent histological analyses of MUB purified from *L. reuteri* have suggested that MUB recognizes terminal sialic acid residues in mucin chains, and thus the distinct aspects of MUB adhesion to mucin are gradually being uncovered [74]. Moreover, chemically synthesized MUB_70_ originating from the MucBP-associated domain (MUBAD), which differs from the *L. reuteri* MUB-encoded MucBP domain, was characterized to bind to sulfated carbohydrates of Muc2-type mucin by using pull-down assays and histochemical techniques [75]. During the onset of colonic mucinous carcinomas, Muc2-type mucin is excessively secreted; this suggests that a potential marker for mucosal cancer could be developed based on the specific binding affinity of MUB_70_.

Mannose-specific adhesin (Msa) is a protein of 1,010 amino acid residues from *Lactobacillus plantarum* WCSF-1 that contains conserved sequences that are highly homologous with sequences of the ConA lectin-like SasA domain and MucBP domain [78]. This suggests that the protein uses a carbohydrate chain containing a mannose as receptor, although biochemical investigations are required to investigate this in the context of mucins.

More recently, genomic analyses identified putative adhesion factors based on the presence of the YSIRK signal, LPXTG motif, or MucBP domains in the sequence. For example, mucus-binding factor (MBF) was identified in *Lactobacillus rhamnosus*, which binds to mucin and extracellular matrix (ECM) proteins including laminin, fibronectin, and collagen IV [79,80], and cell and mucus-binding protein A (CmbA) was identified in *L. reuteri*, which binds to mucin and Caco-2 cells [81]. Almost concurrently, Lar_0958, a previously unidentified protein containing a repeat pattern that differs from known Mub proteins, was discovered in *L. reuteri* JCM1112 [70], and this was the same protein as CmbA [81]. The protein contributes to mucin adhesion and bacterial co-aggregation [70]. All of these factors were analyzed in strains harboring mutant adhesion-factor genes in order to reveal the effects of different genetic mutations on the adhesion of each strain.

**Figure 6:**
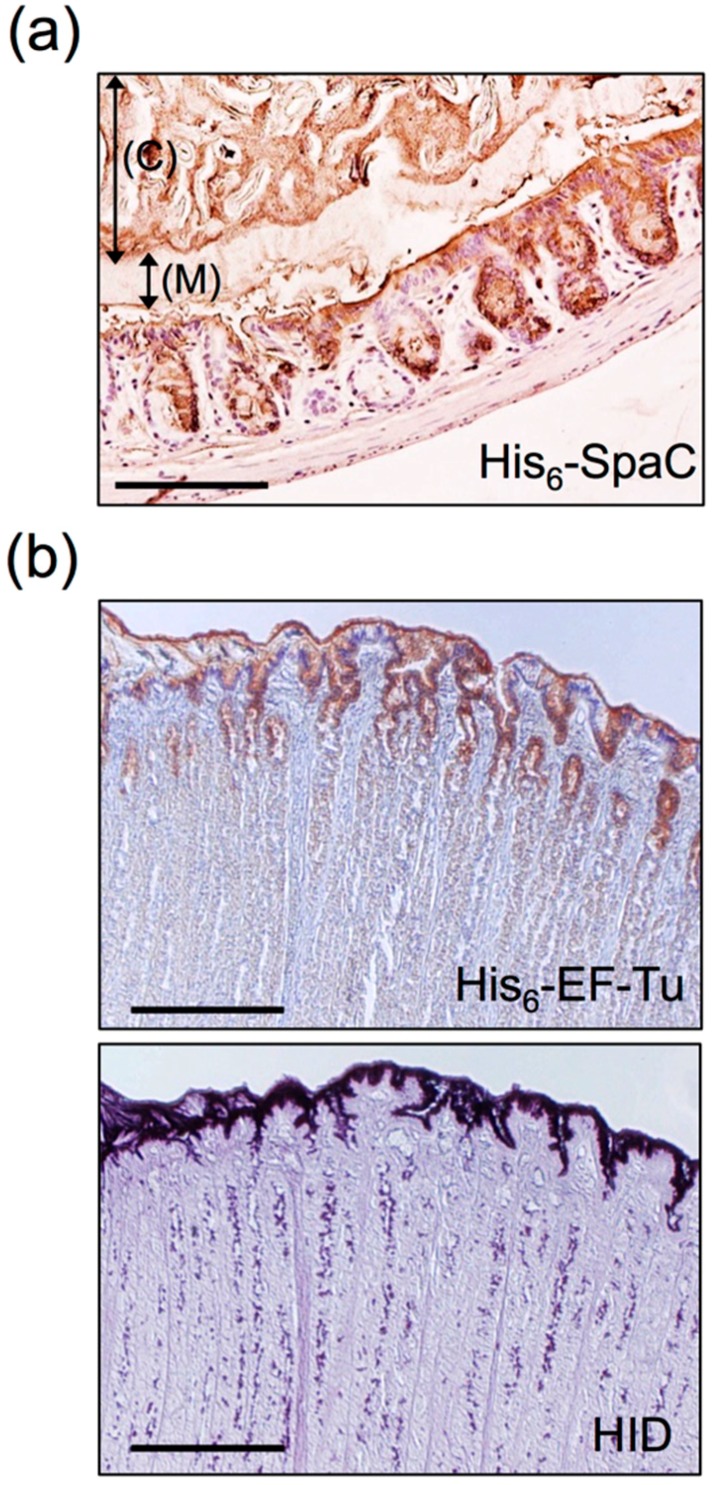
Histochemical staining of the Methacarn-fixed GI mucosal surface with recombinant proteins. **(a)** Binding of His_6_-SpaC was observed on the murine colonic mucosa. Arrows indicate the gut contents (C) and the mucus layer (M). Scale bar: 200 µm. Based on the results of Nishiyama et al. [47]. **(b)** Binding of His_6_-EF-Tu was observed on the porcine gastric mucosal surface (upper panel). These areas were coincident with areas positively stained for high iron diamine (HID) (lower panel). Scale bars: 1000 µm. Based on the results of Nishiyama et al. [86].

Other mucin adhesion factors have been discovered by exploiting genomic information. Pili (fimbriae), for example, were long considered to be a feature unique to pathogenic bacteria. However, after the entire genome of *Lactobacillus johnsonii* NCC533 was sequenced, the existence of a gene cluster encoding pili was reported for the first time in *Lactobacillus* species [82], following which pili were identified through immunoelectron microscopy on the cell surface of *L. rhamnosus* GG [76]. The pili of *L. rhamnosus* are formed by the polymerization of 3 subunits, SpaA, B, and C (called SpaCBA). SpaC, which is present along the whole pilus length, is reported to be involved in mucin binding (Figure 5, right) [76,77,83]. Moreover, we have recently discovered that SpaC possesses lectin-like properties, and this enables SpaC to specifically bind to the galactosyl group located on the non-reducing terminal of mucin [47]. SpaC was also found to specifically bind to the gut contents, gel mucus layer, and intestinal glands in murine colonic mucosa sections (Figure 6a). Comparative genomic analysis of 100 *L. rhamnosus* strains revealed that the production of SpaCBA pili was considerably more prevalent in human isolates than in dairy isolates [84]. Douillard and colleagues also suggested that the lack of the gene cluster encoding SpaCBA pili in most dairy strains reflects a possible niche specialization to a habitat in which pili are not essential and do not offer any benefit for colonization. In addition to the SpaCBA operon, another set of genes for a second type of pilus (called SpaFED) is found in the *L. rhamnosus* GG genome [76]. The SpaFED pilus is produced in *Lactococcus lactis*, with the tip-located SpaF pilin serving as the focal determinant for pilus-mediated cellular interactions with mucins, collagen, and fibronectin, as well as with the Caco-2 and HT-29 intestinal cell lines [85].

Recently, pili have also been shown to be required for adhesion of *Bifidobacterium* for host colonization. The type IVb or so-called tight-adherence pili (Tad pili) from *Bifidobacterium breve* UCC2003 and the sortase-dependent pili from *Bifidobacterium bifidum* PRL2010 have been implicated in GI tract colonization and the persistence of bifidobacteria in the murine gut [28,87]. The fimbria-associated BL0675 subunit (FimA) is found in several *Bifidobacterium longum* subsp. *longum* strains [88,89], and the mucin-binding properties of this protein are affected by genetic polymorphisms [90]. These appendages, which decorate the surface of commensal bacteria, are increasingly regarded as key molecules in the mediation of bacterial adherence to the host mucosal surface.

### 6.2. Moonlighting Proteins

Moonlighting proteins were named based on the term “moonlighting”, which refers to working a side job; these proteins perform two or more functions in addition to their primary (originally identified) function [91]. In lactobacilli, the following proteins have been reported to act as mucin adhesion factors (Table 1): the elongation factor Tu (EF-Tu) [86,92,93,94], glyceraldehyde 3-phosphate dehydrogenase (GAPDH) [95,96,97], the chaperonin GroEL [98], and the ATP-binding cassette (ABC) transporter [99,100,101,102,103]. A few moonlighting proteins have been shown to reside on the cell surface by binding to the cell wall through electrostatic forces. When the extracellular pH becomes weakly alkaline, EF-Tu and GAPDH are completely released from the cell surface [86,104]. These proteins do not contain a typical conserved cell-surface-anchoring motif, and their cell-surface localization is affected by environmental conditions; therefore, these proteins might appear to function as minor adhesion factors, but they exhibit specific interactions with carbohydrate chains.

Mucus adhesion-promoting protein (MapA) from *L. reuteri* 104R [99,100], a 32-kDa mucus- and mucin-binding protein (32-Mmubp) from *L. fermentum* BCS87 [101], and a 29-kDa protein (Lam29) from *L. mucosae* ME-340 [102,103] are components of ABC transporters, but have also been shown to function as mucin- and epithelial cell-adhesion factors. Furthermore, in an experiment conducted using carbohydrate chain probes, GAPDH from *L. plantarum* LA318 was found to exhibit binding activity for GalNAc and galactose, which are present on A and B blood-group antigens [95,96]. Lam29 was also reported to bind to blood-group antigens [102,103]. These adhesion factors might mediate the adhesion of certain *Lactobacillus* strains to mucin carbohydrate chains through blood-group antigens, as described in Section 5.

Moreover, we discovered that under acidic conditions, EF-Tu from *L. reuteri* JCM1081 exhibited binding activity for acidic oligosaccharides purified from sulfated glycolipids or mucin [86,92]. Furthermore, the reaction site of high iron diamine (HID) stain, which is used for staining sulfated groups, resembled that of the EF-Tu protein in tissue slices of porcine gastric mucosal surface (Figure 6b). Interestingly, EF-Tu showed little or no affinity for negatively charged sialic acid, but specifically bound to mucin’s sulfate group or sulfated blood-group antigen [86]. Notably, these pH-dependent binding properties of EF-Tu were similar to those described by Granato et al. [93], who found that the EF-Tu protein of *L. johnsonii* NCC533 bound efficiently to mucin and also to Caco-2 and HT-29 intestinal cell lines under acidic conditions, but not under neutral conditions. The amino acid sequence of EF-Tu shared high identity with that of *L. johnsonii* NCC533. Thus, the binding of EF-Tu to the mucosal surface might be mediated by sulfated carbohydrates in other *Lactobacillus* species. Furthermore, because sulfated carbohydrates represent one of the receptors involved in *H. pylori* infection of a host [57], in vitro and in vivo experiments were conducted by exploiting EF-Tu’s binding specificity in order to test for the hypothesized competitive inhibition of the adhesion between *H. pylori* and EF-Tu [92,105].

The production of genetic mutants, however, will not be a simple task, because several moonlighting proteins are essential for growth of lactobacilli, and this makes evaluation of their contribution to *Lactobacillus* adhesive properties challenging. Although lectin-like domains or similar characteristic sequences in these proteins have not yet been reported, further research is expected to unravel the mystery of why these proteins exhibit specific binding activity toward carbohydrate chains.

## 7. Conclusions

Whole-genome sequencing of bacteria and metagenomic analyses of microbiota using comprehensive genomic analytical techniques have allowed us to piece together the symbiotic relationship between the host and the microorganisms within the complex ecosystem that is the human body. Therefore, the existence of a specific yet stable microbiota is becoming increasingly clear. Adhesion of *Lactobacillus* to the mucosal surface has been considered one of the initial events in the successful colonization of the host GI tract. There is considerable interest in determining how *Lactobacillus* can colonize and coexist with their host, and a substantial amount of research has recently been focused on whether this ability depends on specific host–LAB interactions.

As discussed in this review, lactobacilli exhibit various adhesive properties on mucin and mucin carbohydrate chains based on a wide variation of molecular structures. This implies that *Lactobacillus* adapt to the constantly changing intestinal environment of the host, and further suggests that adhesion factors of *Lactobacillus* possess specific binding affinities that allow them to advantageously colonize the host while concurrently avoiding competition with other bacteria. Therefore, findings on lactobacilli adhesion factors for mucin provide crucial insights that enhance our understanding of the unique characteristics of *Lactobacillus*. Currently, findings on lactobacilli adhesion mechanisms have halted at the level of specific strains, and therefore comparative analyses of these strains is a key direction for further research. Moreover, despite the increasing knowledge regarding adhesion factors, several binding epitopes on the carbohydrate chains remain unresolved, and these will be targets of future studies. Deepening our understanding of the close relationship between *Lactobacillus* and mucin is expected to provide crucial findings that will help us to propose additional specific applications of *Lactobacillus*, and elucidate at least one part of the host-adhesion mechanism.

## Figures and Tables

**Table 1:** Mucin adhesion factors in *Lactobacillus* species.

Adhesion Factor	Species	Anchoring Type	Reference
CmbA/Lar_0958	*L. reuteri* ATCC PTA 6475, JCM1112	LPXTG	[70,81]
EF-Tu	*L. johnsonii* NCC533	Anchorless	[86,92,93,94]
	*L. reuteri* JCM1081		
	*L. plantarum* strains		
GAPDH	*L. acidophilus* La14	Anchorless	[95,96,97]
	*L. plantarum* La318		
GroEL	*L. johnsonii* NCC533	Anchorless	[98]
Lam29	*L. mucosae* ME-340	Membrane bound	[102,103]
MapA	*L. reuteri* 104R	Membrane bound	[99,100]
MBF	*L. rhamnosus* GG, FSMM22	LPXTG	[79,80]
Msa	*L. plantarum* WCFS-1	LPXTG	[78]
Mub (Mub family)	*L. acidophilus* NCFM	LPXTG	[68,69,70,71,73,74]
	*L. reuteri* strains		
Pili	*L. rhamnosus* GG	LPXTG (Fimbriae type)	[47,76,77,83,85]
32-Mmubp	*L. fermentum* BCS87	Membrane bound	[101]
